# Curcumin Prevents Replication of Respiratory Syncytial Virus and the Epithelial Responses to It in Human Nasal Epithelial Cells

**DOI:** 10.1371/journal.pone.0070225

**Published:** 2013-09-18

**Authors:** Kazufumi Obata, Takashi Kojima, Tomoyuki Masaki, Tamaki Okabayashi, Shinichi Yokota, Satoshi Hirakawa, Kazuaki Nomura, Akira Takasawa, Masaki Murata, Satoshi Tanaka, Jun Fuchimoto, Nobuhiro Fujii, Hiroyuki Tsutsumi, Tetsuo Himi, Norimasa Sawada

**Affiliations:** 1 Department of Otolaryngology, Sapporo Medical University School of Medicine, Sapporo, Japan; 2 Department of Pathology, Sapporo Medical University School of Medicine, Sapporo, Japan; 3 Department of Cell Science, Research Institute of Frontier Medicine, Sapporo Medical University School of Medicine, Sapporo, Japan; 4 Research Institute for Microbial Diseases, Osaka University, Suita, Japan; 5 Department of Microbiology, Sapporo Medical University School of Medicine, Sapporo, Japan; 6 Department of Pediatrics, Sapporo Medical University School of Medicine, Sapporo, Japan; University of North Carolina at Chapel Hill, United States of America

## Abstract

The human nasal epithelium is the first line of defense during respiratory virus infection. Respiratory syncytial virus (RSV) is the major cause of bronchitis, asthma and severe lower respiratory tract disease in infants and young children. We previously reported in human nasal epithelial cells (HNECs), the replication and budding of RSV and the epithelial responses, including release of proinflammatory cytokines and enhancement of the tight junctions, are in part regulated via an NF-κB pathway. In this study, we investigated the effects of the NF-κB in HNECs infected with RSV. Curcumin prevented the replication and budding of RSV and the epithelial responses to it without cytotoxicity. Furthermore, the upregulation of the epithelial barrier function caused by infection with RSV was enhanced by curcumin. Curcumin also has wide pharmacokinetic effects as an inhibitor of NF-κB, eIF-2α dephosphorylation, proteasome and COX2. RSV-infected HNECs were treated with the eIF-2α dephosphorylation blocker salubrinal and the proteasome inhibitor MG132, and inhibitors of COX1 and COX2. Treatment with salubrinal, MG132 and COX2 inhibitor, like curcumin, prevented the replication of RSV and the epithelial responses, and treatment with salubrinal and MG132 enhanced the upregulation of tight junction molecules induced by infection with RSV. These results suggest that curcumin can prevent the replication of RSV and the epithelial responses to it without cytotoxicity and may act as therapy for severe lower respiratory tract disease in infants and young children caused by RSV infection.

## Introduction

Respiratory syncytial virus (RSV) is a negative-stranded RNA virus in the genus Pneumovirus, family Paramyxoviridae and is the major cause of bronchitis, asthma and severe lower respiratory tract disease in infants and young children [1]. There is no effective vaccine, and the use of passive RSV-specific antibodies is limited to high-risk patients [2].

The envelope of RSV contains three transmembrane surface proteins, the fusion F glycoprotein, attachment G glycoprotein and small hydrophobic protein (SH protein) [3], [4]. Recently, the fusion envelope glycoprotein of RSV was reported to bind specifically to nucleolin at the apical cell surface for entering through the host-cell and nucleolin was found to be a functional cellular receptor for RSV [5]. Furthermore, RSV has M2-1 protein, which induces transcriptional processivity and is an anti-termination factor [6], and M2-1 protein induces the production of cytokines and chemokines via activation of nuclear factor kappa B (NF-κB) [7]. RSV also induces and activates protein kinase R (PKR), a cellular kinase relevant to limiting viral replication, which regulates the activation of a translation initiation factor, the α subunit of eukaryotic translation initiation factor 2 (eIF-2α) [8]–[10].

On the other hand, it is thought that RSV replicates in the airway mucosa, where it may produce uncomplicated upper respiratory infection or spread distally to the lower airways, producing more severe lower respiratory tract infection. We recently reported that, in human nasal epithelial cells (HNECs), the replication and budding of RSV and the epithelial responses, including the release of proinflammatory cytokines and the epithelial barrier function of tight junctions, were regulated via the protein kinase Cδ (PKCδ)/hypoxia-inducible factor-1alpha (HIF-1α)/NF-κB pathway [11]. It is known that RSV affects NF-κB-dependent expression of various genes [12]. Furthermore, the proinflammatory cytokines IL-8 and TNF-α and chemokines RANTES (CCL5) and CXCL10 induced by RSV are regulated via an NF-κB pathway [13]–[15]. This NF-κB pathway plays an important role in RSV-induced respiratory pathogenesis. Furthermore, in HNECs, RSV induces cytosolic pattern recognition receptors (PRRs), retinoic acid-inducible gene I (RIG-I), melanoma differentiation-associated gene 5 (MDA5), and interferon (IFN)-λ, but not IFN-α/β, and the IFN-λ contributes to the main first line of defense via a RIG-I-dependent pathway against RSV infection [16].

The airway epithelium, particularly the nasal epithelium, is the first line of defense against respiratory virus infection [17]. The epithelial barrier of the airway is regulated in large part by the apicalmost intercellular junctions, referred to as tight junctions [18]. Tight junctions are formed by not only the integral membrane proteins claudins, occludin, tricellulin, JAMs (junctional adhesion molecules) and CAR (coxsackie and adenovirus receptor), but also many peripheral membrane proteins, including scaffold PDZ-expression proteins and cell polarity molecules [19]–[22]. Moreover, some tight junction molecules are thought to be targets or receptors of viruses such as claudin-1 and occludin as coreceptors of HCV, JAM as a reovirus receptor, and CAR as a coxsackie and adenovirus receptor [23]. In RSV-infected HNECs, expression of claudin-4 and occludin is upregulated together with the barrier function via a PKCδ/HIF-1α/NF-κB pathway, whereas claudin-4 and occludin are not receptors of RSV in HNECs, as revealed by experiments using siRNAs [11].

Curcumin [1,7-bis (4-hydroxy-3-methoxyphenyl)-1,6-heptadiene-3,5dione], is a major phenolic compound from the rhizome of the plant *Curcuma longa*, and has various functions, including antiviral, anti-inflammatory, antioxidant and anticancer effects [24]–[26]. It is thought that the effects of curcumin are in part caused by inhibition of NF-κB, since curcumin prevents the entry of NF-κB into the nucleus [27]. Furthermore, curcumin is not only an inhibitor of NF-κB but also a potent inhibitor of proteasome, cyclooxygenase-2 (COX2), lipooxygenase, ornithine decarboxylase, c-Jun N-terminal kinase and protein kinase C [28]–[30]. Curcumin modulates eIFs, which play important roles in translation initiation, cell growth and proliferation [25]. Curcumin increases the epithelial barrier by enhancing tight junctions and adherens junctions [31], [32].

In this study, we confirmed that, in a model of RSV-infected HNECs [11], the NF-κB inhibitor curcumin could prevent the replication, assembly and budding of RSV, and the epithelial responses to the virus indicated as expression of RIG-I and MDA5, release of TNFα and RANTES. Furthermore, to investigate the detailed mechanisms on the effects of curcumin in RSV-infected HNECs, other NF-κB inhibitors, the eIF2α dephosphorylation blocker salubrinal and the proteasome inhibitor MG132, and COX1 and COX2 inhibitors were also used. Because curcumin has mutipotential inhibitory effects of eIF-2α dephosphorylation, and activities of proteasome and COX2 [28]–[30]. We found that in HNECs, curcumin, an inhibitor of NF-κB, eIF-2α dephosphorylation, proteasome and COX2, prevented the replication and budding of RSV and inhibited the upregulation of both expression of RIG-I and MDA5 and release of TNFα and RANTES induced by RSV infection.

## Materials and Methods

### Reagents and inhibitors

Curcumin was purchased from Cayman Chemical Corporation (Ann Arbor, MI). IMD0354, pyrollidine dithiocarbamate (PDTC) and 4′,6-diamidino-2-phenylindole dihydrochloride (DAPI) were purchased from Sigma-Aldrich (St. Louis, MO). MG132, the COX1 inhibitor (FR122047), and COX2 inhibitor were purchased from Calbiochem Novabiochem Corporation (San Diego, CA). Salubrinal was purchased from Tocris Bioscience (St. Louis, MO). Alexa 488 (green) and Alexa 594 (red) conjugated anti-mouse and anti-rabbit IgG antibodies were purchased from Molecular Probes, Inc. (Eugene, OR). HRP-conjugated polyclonal goat anti-rabbit immunoglobulins were purchased from Dako A/S (Glostrup, Denmark). The ECL Western blotting system was obtained from GE Healthcare UK, Ltd. (Buckinghamshire, UK).

### Cell culture and treatments

The cultured HNECs were derived from mucosal tissues of patients with hypertrophic rhinitis or chronic sinusitis who underwent inferior turbinectomy at Sapporo Medical University, the Sapporo Hospital of Hokkaido Railway Company, or the KKR Sapporo Medical Center Tonan Hospital. The study protocol was approved by the Institutional Review Board of these Hospital, and all subjects gave informed written consent before enrollment in this study.

The methods for primary culture of human nasal epithelial cells were as reported previously [33]. Some primary cultured HNECs were transfected with the catalytic component of telomerase, the human catalytic subunit of the telomerase reverse transcriptase (hTERT) gene, as described previously [11], [34]. The cells were plated on 35-mm or 60-mm culture dishes (Corning Glass Works, Corning, NY), which were coated with rat tail collagen (500 μg of dried tendon/ml 0.1% acetic acid). The cells were cultured in serum-free bronchial epithelial cell basal medium (BEBM, Lonza Walkersville, Inc.; Walkersville, MD) supplemented with bovine pituitary extract (1% v/v), 5 μg/ml insulin, 0.5 μg/ml hydrocortisone, 50 μg/ml gentamycin, 50 μg/ml amphotericin B, 0.1 ng/ml retinoic acid, 10 μg/ml transferrin, 6.5 μg/ml triiodothyronine, 0.5 μg/ml epinephrine, 0.5 ng/ml epidermal growth factor (Lonza Walkersville, Inc.), 100 U/ml penicillin and 100 μg/ml streptomycin (Sigma-Aldrich) and incubated in a humidified, 5% CO_2_:95% air incubator at 37°C. In this experiment, 2nd and 3rd passaged cells were used.

The A549 human lung adenocarcinoma epithelial cell line (ATCC, Manassas, VA) was maintained in RPMI 1640 medium with 10% fetal bovine serum (FBS).

Human RSV was grown in the human laryngeal carcinoma cell line HEp-2. For infection, HNECs at 80% confluence were adsorbed at an RSV multiplicity of infection (MOI) of 1 for 60 min at 37°C. After adsorption, the viral solutions were removed and the cells were rinsed twice with growth medium and incubated. The virus titers in the supernatant were determined by a plaque-forming assay with HEp-2 cells. Expression of RSV mRNA was confirmed by reverse transcription-PCR (RT-PCR).

Some cells were incubated in a 2% CO_2_:2% O_2_ incubator balanced by nitrogen. They were pretreated with 0.1–10 µg/ml curcumin, 0.1–10 µg/ml IMD0354, 0.1–10 µg/ml PDTC, 0.1–10 µg/ml MG132, 0.1–50 µg/ml salubrinal, 0.1–10 µg/ml COX1 inhibitor and 0.1–10 µg/ml COX2 inhibitor at 30 min before RSV infection.

### MTT assay

The cells plated on 24-well tissue culture plates (BD Labware, Flanklin Lakes, NJ) were treated with 0.1–50 μg/ml HWE for 24 h. The cell survival was evaluated with a colorimetric assay using an MTT Cell Growth Assay Kit (Millipore, Billerica, MA) according to the manufacturer's recommendations. The ratio of absorbance was calculated and presented as the mean ± SEM of triplicate experiments.

### GeneChip analysis

Microarray slides were scanned using 3D-GENE human 25k. (TORAY, Tokyo, Japan) and microarray images were automatically analyzed using AROS^TM^, version 4.0 (Operon Biotechnologies, Tokyo, Japan).

### Western blot analysis

The hTERT-transfected HNECs were scraped from a 60 mm dish containing 300 μl of buffer (1 mM NaHCO3 and 2 mM phenylmethylsulfonyl fluoride), collected in microcentrifuge tubes, and then sonicated for 10 s. The protein concentrations of the samples were determined using a BCA protein assay reagent kit (Pierce Chemical Co., Rockford, IL). Aliquots of 15 μl of protein/lane for each sample were separated by electrophoresis in 4–20% SDS polyacrylamide gels (Daiichi Pure Chemicals Co., Tokyo, Japan), and electrophoretically transferred to a nitrocellulose membrane (Immobilon, Millipore Co., Bedford, UK). The membrane was saturated for 30 min at room temperature with blocking buffer (25 mM Tris, pH 8.0, 125 mM NaCl, 0.1% Tween 20, and 4% skim milk) and incubated with anti-phospho-NFκB, anti- NFκB, anti-phospho-eIF-2α, anti- eIF-2α, anti-phospho-IκB, anti-IκB, anti-PKR, anti-nucleolin, anti-phospho-PERK, anti-actin, anti-occludin, anti-claudin-4, anti-M2-1 protein, and anti-G protein antibodies (Table 1) at room temperature for 1 h. Then it was incubated with HRP-conjugated anti-mouse and anti-rabbit IgG antibodies at room temperature for 1 h. The immunoreactive bands were detected using an ECL Western blotting system.

**Table 1:** Antibodies.

Antibody	Type	Dilution		Source
		IC	WB	
Claudin-4	pAb	1:100	1:1000	Zymed Laboratories (San Francisco, CA)
Occludin	pAb	1:100	1:1000	Zymed Laboratories
G and F proteins	mAb	1:100	1:2000	Tsutsumi et al., 1989
M2-1 protein	mAb		1:2000	Tsutsumi et al., 1989
Phospho-NF-κB	pAb		1:500	Cell signaling Technology (Santa Cruz, CA)
NF-κB	pAb		1:1000	Cell signaling Technology
Phospho-eIF-2α	pAb		1:500	Cell signaling Technology
eIF-2α	pAb		1:1000	Cell signaling Technology
PKR	pAb		1:1000	Cell signaling Technology
Nucleolin	pAb		1:1000	Abcam (Cambridge, MA)
Actin	pAb		1:1000	Sigma-Aldrich (St. Louis, MO)

### RNA isolation, RT-PCR and real-time PCR analysis

Total RNA was extracted and purified using TRIzol (Invitrogen, Carlsbad, CA). One microgram of total RNA was reverse-transcribed into cDNA using a mixture of oligo (dT) and Superscript II reverse transcriptase according to the manufacturer's recommendations (Invitrogen). Synthesis of each cDNA was performed in a total volume of 20 µl for 50 min at 42°C and terminated by incubation for 15 min at 70°C. PCR was performed in a 20 µl total mixture containing 100 pM primer pairs, 1.0 µl of the 20 µl total RT product, PCR buffer, dNTPs and Taq DNA polymerase according to the manufacturer's recommendations (Takara, Kyoto, Japan). Amplifications were for 25–35 cycles depending on the PCR primer pair with cycle times of 15 s at 96°C, 30 s at 55°C and 60 s at 72°C. Final elongation time was 7 min at 72°C. Seven microliters of the total 20 µl PCR product was analyzed by 1% agarose gel electrophoresis with ethidium bromide staining and standardized using a GeneRuler TM 100 bp DNA ladder (Fermentas, Ontario, Canada). To provide a quantitative control for reaction efficiency, PCR reactions were performed with primers coding for the housekeeping gene glyceraldehyde-3-phosphate dehydrogenase (G3PDH). Primers used to detect G3PDH and claudin-4, occludin, RSV G protein, RIG-I, and MDA5 are indicated in Table 2.

**Table 2:** Primers of RT-PCR.

Gene	Forward primer	Reverse primer	Product size (base pairs)
G protein	GGGGCAAATGCAAACATGT	GGTATTCTTTTGCATATAGC	621
RIG-I	CCTATGCAGCTCCGCCTCGC	GCCACGGAACCAGCCTTCCT	360
MDA5	GCAAGAGCATCCCCGGAGCC	TCGTGGCCCCTCCAACACCA	601
G3PDH	ACCACAGTCCATGCCATCAC	TCCACCACCCTGTTGCTGTA	452

Real-time PCR detection was performed using a TaqMan Gene Expression Assay kit with a StepOnePlus^TM^ real-time PCR system (Applied Biosystems, Foster City, CA). The amount of 18S ribosomal RNA (rRNA) (Hs99999901) mRNA in each sample was used to standardize the quantities of the following mRNAs: claudin-4 (Hs00533616), occludin (Hs00170162), and RIG-I (Hs00204833). The relative mRNA-expression levels between the control and treated samples were calculated by the difference of the threshold cycle (comparative C_T_ [ΔΔC_T_] method) and presented as the average of triplicate experiments with a 95% confidence interval.

### Enzyme-linked immunosorbent (ELISA) assay

The concentrations of human TNFα and RANTES in cell culture supernatants of hTERT-transfected HNECs at 24–72 h after treatment with RSV were measured using ELISA kits for human TNFα (R&D Systems, Minneapolis, MN) and RANTES (PeproTech EC, London, UK) according to the manufacturers' instructions.

### Immunocytochemistry

hTERT-transfected HNECs grown in 35 mm glass-coated wells (Iwaki, Chiba, Japan) were fixed with cold acetone and ethanol (1∶1) at –20°C for 10 min. After rinsing in PBS, the cells were incubated with anti-G, F, and M2-1 proteins, and anti-occludin, anti-claudin-4 antibodies (Table 1) overnight at 4°C. Alexa Fluor 488 (green)-conjugated anti-rabbit IgG and Alexa Fluor 592 (red)-conjugated anti-mouse IgG (Invitrogen) were used as secondary antibodies. The specimens were examined and photographed with an Olympus IX 71 inverted microscope (Olympus Co., Tokyo, Japan) and a confocal laser scanning microscope (LSM510, Carl Zeiss, Jena, Germany).

### Scanning electron microscopy (SEM)

Cells grown on coated coverslips were fixed with 2.5% glutaraldehyde/0.1 M PBS (pH 7.3) overnight at 4°C. After several rinses with PBS, the cells were postfixed in 1% osmium tetroxide at 4°C for 3 h and then rinsed with distilled water, dehydrated in a graded ethanol series, and freeze-dried. The specimens were sputter-coated with platinum and observed with a scanning electron microscope (S-4300, Hitachi, Tokyo, Japan) operating at 10 kV.

### Measurement of transepithelial electrical resistance (TER)

hTERT-transfected HNECs were cultured to confluence on inner chambers of 12-mm Transwell inserts with 0.4-µm pore-size filters (Corning Life Sciences). TER was measured using the CellZscope (Nanoanalytics, Münster) adjusted to 37°C. The values were expressed in standard units of ohms per square centimeter and presented as the mean ± S.D. For calculation, the resistance of blank filters was subtracted from that of filters covered with cells.

### Data analysis

Signals were quantified using Scion Image Beta 4.02 Win (Scion Co., Frederick, MA). Each set of results shown is representative of at least three separate experiments. Results are given as means ± SEM. Differences between groups were tested by a post-hoc test and an unpaired two-tailed Student's t test.

## Results

### Inhibitors of NF-κB prevent replication of RSV in human nasal epithelial cells (HNECs) infected with RSV

**Figure 1:**
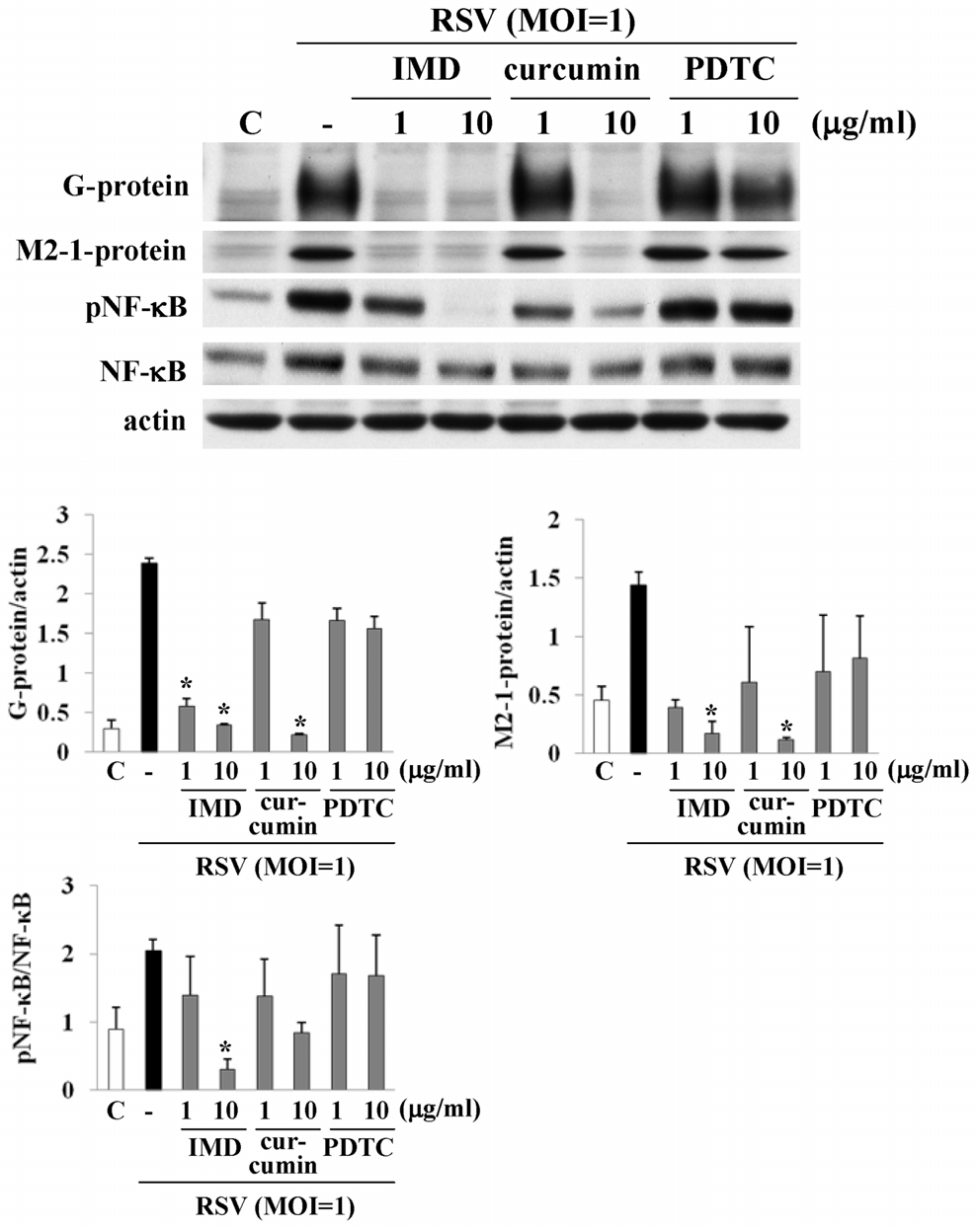
Western blotting for G and M2-1 proteins, phospho-NF-κB and NF-κB in HNECs pretreated with IMD0352, curcumin and PDTC before infection with RSV at an MOI of 1. The corresponding expression levels are shown as error bar graphs. Data are means ± SEM. *p<0.05 versus only RSV. IMD: IMD0354, PDTC: pyrrolidine dithiocarbamate.

We previously found that the NF-κB inhibitor IMD0354 prevented replication of RSV in an established RSV-infected model using hTERT-transfected HNECs [11]. In the present study, we investigated whether other NF-κB inhibitors, curcumin and PDTC, could prevent replication of RSV compared to IMD-0354. When HNECs were pretreated with IMD0354, curcumin and PDTC at 1 and 10 µg/ml 30 min before infection with RSV at an MOI of 1 for 24 h, 1 and 10 µg/ml IMD0354 and 10 µg/ml curcumin, but not PDTC, prevented production of RSV/G- and M2-1-proteins, which indicated the replication of RSV, together with a decrease of phospho-NF-κB in Western blotting (Figure 1).

### Effects of curcumin on cell viability, expression of NF-κB, claudin-4 and occludin in HNECs

**Figure 2:**
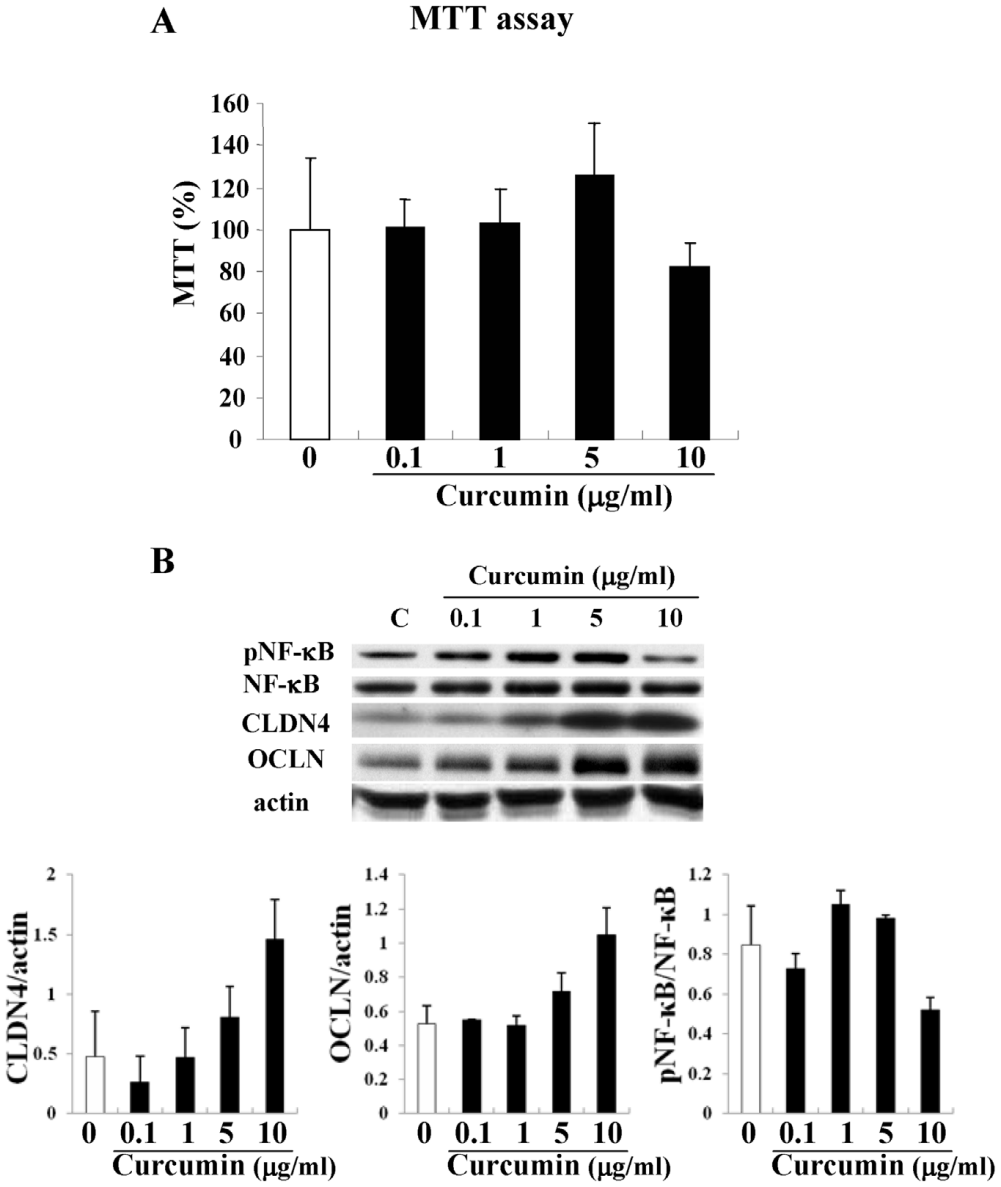
Survival rates of HNECs after treatment with 0.1–10 μg/ml curcumin for 24 h were determined by MTT assay. Error bars represent means ± SEM. n = 3 *p<0.05 versus control. Western blotting for phospho-NF-κB, NF-κB, claudin-4 and occludin in HNECs treated with 0.1–10 μg/ml curcumin. The corresponding expression levels are shown as error bar graphs. Data are means ± SEM. CLDN: claudin, OCLN: occludin.

To investigate the effects of curcumin on cell viability of HNECs, we measured the survival rates of HNECs after treatment with 0.1–10 μg/ml curcumin for 24 h by MTT assay. As shown in Figure 2A, curcumin did not affect the cell viability even at high concentrations. When HNECs were treated with 0.1–10 μg/ml curcumin for 24 h, in Western blotting downregulation of phospho-NF-κB was observed at 10 μg/ml curcumin and upregulation of claudin-4 and occludin was observed from 5 μg/ml curcumin (Figure 2B).

### Gene expression changes in HNECs infected with RSV with and without curcumin

As epithelial cell responses to RSV infection, expression of tight junction proteins claudin-4 and occludin, and production of proinflammatory cytokines IL-8 and TNFα were upregulated in HNECs infected with RSV [11]. To investigate whether curcumin affected not only replication of RSV but also the epithelial cell responses, we first performed GeneChip analysis of HNECs infected with RSV with and without 5 µg/ml curcumin, and selected gene probes that were regulated more or less than 2-fold compared to the controls and the RSV infection (Table 3).

**Table 3:** List of gene probes, which are changed more or less than 2 fold to the control vs RSV-infected hTERT-HNECs vs RSV-infected hTERT-HNECs pretreated with 5 µg/ml curcumin.

Gnen name	ID	Gene Bank ID	Fold-change	
			Control vs RSV	RSV vs RSV+curcumin
CLDN1CLDN3CLDN4CLDN9CLDN12OCLNCGN(Cingulin)BAIAP1(MAGI-1)	H200001413opHsV0400000648H300004950H200009827H200026459opHsV0400004868H300009163H300019020	NM_021101NM_001306NM_001305NM_020982NM_012129NM_002538NM_004742	>2>4>2>4	>2>2>2>4<4
DDX58(RIG-I)IFIH1(MDA5)	H200013521H200009565	NM_014314NM_022168	>8>4	<8<2
IL-28AIL-23AIL-11	opHsV0400006650H200010868H200000450	NM_172138;NM_172138NM_016584	>2>8>2	<2<2<4
COX1COX2COX3	opHsV0400005827opHsV0400005844opHsV0400005879	NM_496332	>2>2	<4<4<4

In HNECs infected with RSV without curcumin, upregulation of tight junction molecules claudin-1, −3, −4, −9, and −12, occludin, cingulin, and MAGI-1, the pattern recognition receptors retinoic acid-inducible gene-I (RIG-I), melanoma differentiation-associated gene 5 (MDA5), proinflammatory cytokines IL-28A, IL-23A and IL-11, and cyclooxygenase (COX)1 and COX3 was observed compared to the control. In HNECs infected with RSV with 5 µg/ml curcumin, upregulation of claudin-1, −4, and −12, occludin and cingulin, and downregulation of MAGI-1, RIG-I, MDA5, IL-28A, IL-23A, IL-11, COX1, COX2 and COX3 were observed compared to the cells with RSV infection only.

### Curcumin affects replication of RSV, expression of claudin-4 and occuldin, barrier function, formation of virus filaments, virus budding, and production of proinflammatory cytokines in HNECs infected with RSV

To determine whether curcumin affected replication of RSV, expression of claudin-4 and occludin, barrier function, formation of virus filaments, virus budding, and production of proinflammatory cytokines [11], HNECs were pretreated with 0.1–10 µg/ml curcumin 30 min before infection with RSV at an MOI of 1 for 24 h.

**Figure 3:**
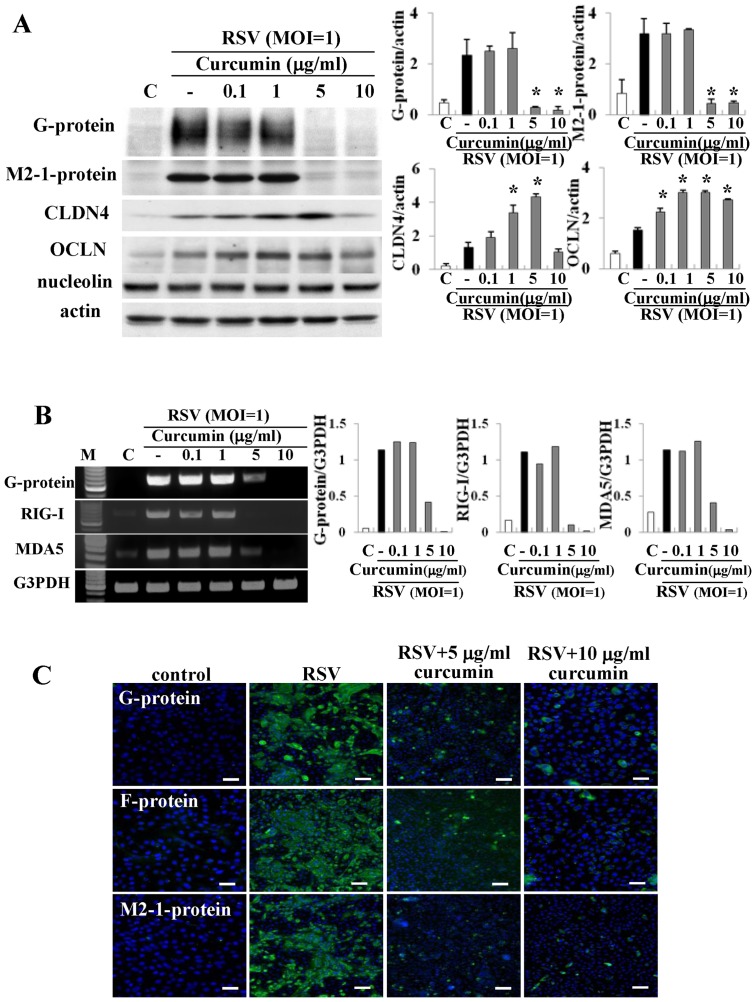
Western blotting (A) for G and M2-1 proteins, claudin-4, occludin and nucleolin, RT-PCR (B) for G protein, RIG-I and MDA5 in HNECs pretreated with 0.1–10 μg/ml curcumin before infection with RSV at an MOI of 1. The corresponding expression levels are shown as error bar graphs. Data are means ± SEM. *p<0.05 versus only RSV. Immunostaining (C) for G, F and M2-1-proteins in HNECs pretreated with 5 and 10 μg/ml curcumin before infection with RSV at an MOI of 1. CLDN: claudin, OCLN: occludin. Bars: 40 μm.

**Figure 4:**
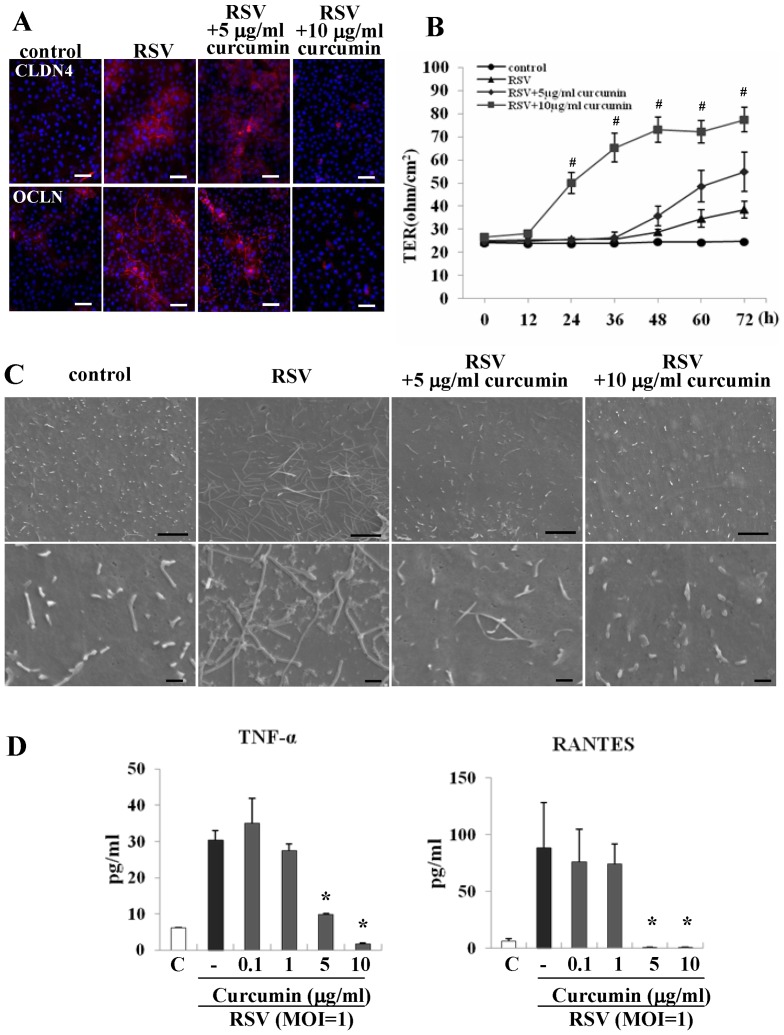
Immunostaining (A) for claudin-4 and occludin, barrier function measured as TER (B) and SEM image (C) in HNECs pretreated with 5 and 10 μg/ml curcumin before infection with RSV at an MOI of 1. ELISA (D) for TNFα and RANTES from HNECs pretreated with 0.1–10 μg/ml curcumin before infection with RSV at an MOI of 1. Error bars represent means ± SEM. n = 3 ^#^p<0.05 versus control. *p<0.05 versus RSV. CLDN: claudin, OCLN: occludin. Bars: A = 40 μm, C = 1 μm.

Expression of G and M2-1 proteins after infection with RSV was inhibited from 5 µg/ml curcumin as detected by Western blotting (Figure 3A). The upregulation of claudin-4 and occludin after infection with RSV was enhanced by 1 and 5 µg/ml curcumin and was prevented by 10 µg/ml curcumin (Figure 3A). No change of RSV coreceptor nucleolin expression was observed in HNECs transfected with RSV with or without curcumin (Figure 3A). In RT-PCR, upregulation of mRNAs of G protein, RIG-I and MDA5 after infection with RSV was inhibited from 5 µg/ml crucumin (Figure 3B). In immunocytochemistry, expression of G, F and M2-1 proteins after infection with RSV was markedly inhibited from 5 µg/ml curcumin, whereas upregulation of claudin-4 and occludin at the membranes after infection with RSV was inhibited by 10 µg/ml curcumin (Figure 3C and 4A). In the barrier function, upregulation of transepithelial electrical resistance (TER) values after infection with RSV was enhanced from 5 µg/ml curcumin (Figure 4B). In SEM, formation of virus filaments and small membranous structures at the surfaces of HNECs after infection with RSV, was inhibited from 5 µg/ml curcumin (Figure 4C). In ELISA, upregulation of TNFα and RANTES production after infection with RSV was significantly inhibited from 5 µg/ml curcumin (Figure 4D).

### Curcumin does not affect replication of RSV in A549 cells infected with RSV

**Figure 5:**
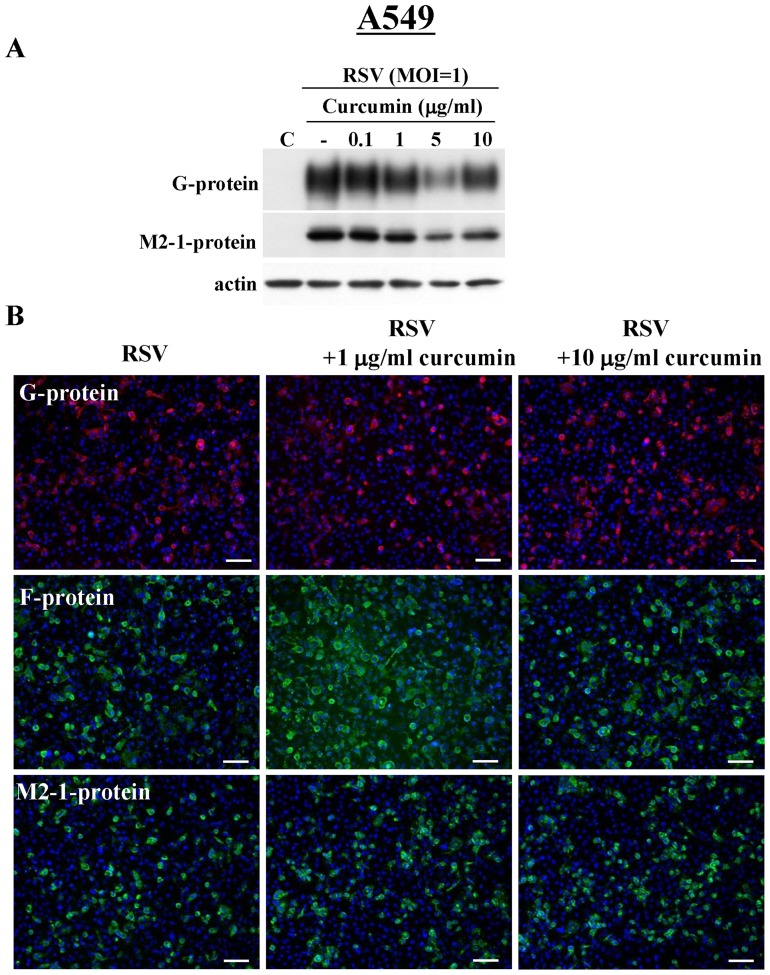
Western blotting (A) for G and M2-1 proteins in A549 cells pretreated with 0.1–10 μg/ml curcumin before infection with RSV at an MOI of 1. Immunostaining (B) for G, F and M2-1 proteins in A549 cells pretreated with 5 and 10 μg/ml curcumin before infection with RSV at an MOI of 1. Bars: 40 μm.

We investigate whether curcumin affect replication human lung adenocarcinoma cell line A549 infected with RSV. In Western blotting, expression of RSV/G and M2-1 proteins after infection with RSV was not inhibited until 10 µg/ml curcumin (Figure 5A). In immunocytochemistry, expression of G, F and M2-1 proteins after infection with RSV was not inhibited until 10 µg/ml curcumin (Figure 5B). In A549 cells, upregulation of claudin-4, occludin and TER was not observed after infection with RSV (data not shown).

### Curcumin affects phospho-NF-κB, phospho-eIF2α and PKR in HNECs infected with RSV

**Figure 6:**
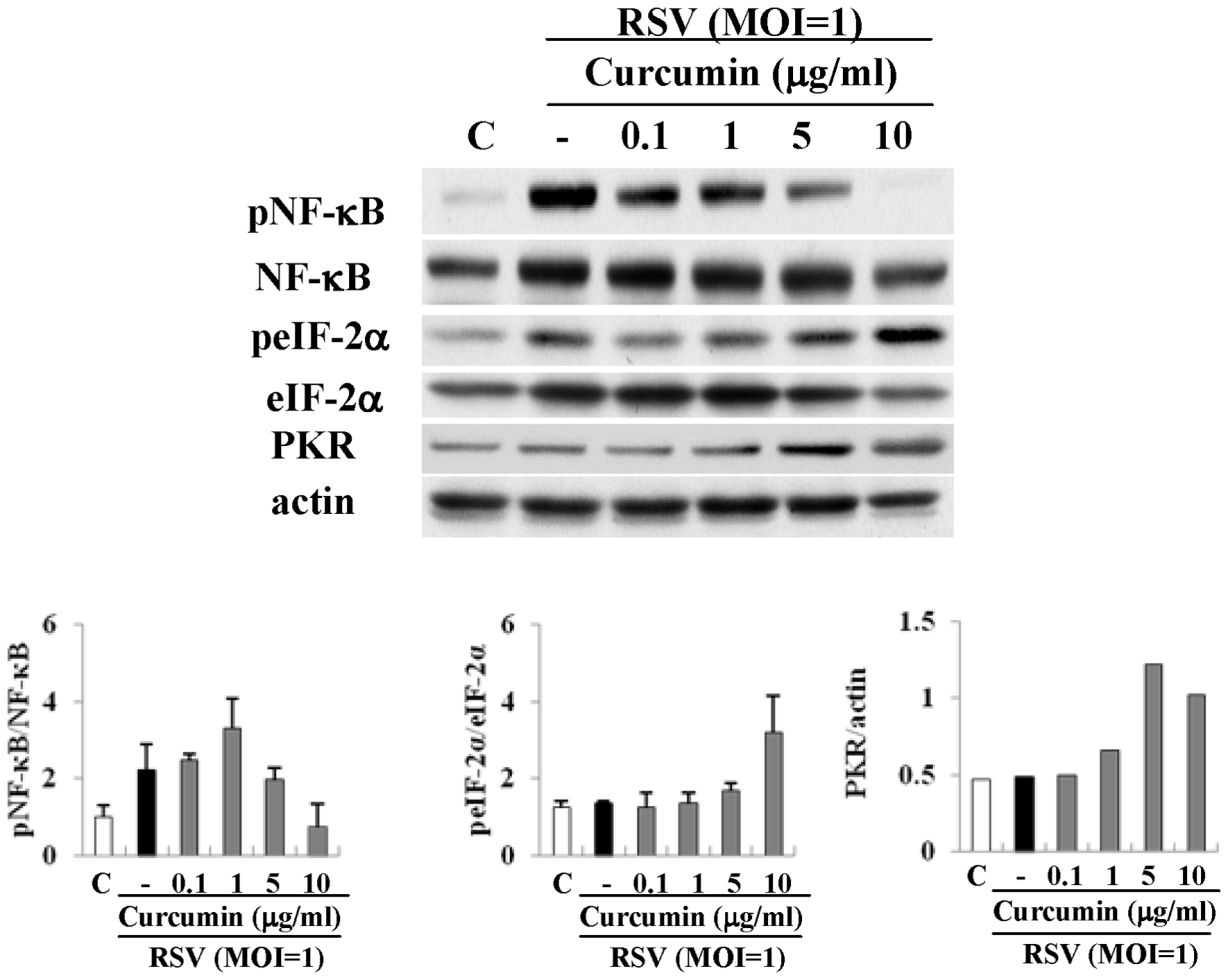
Western blotting for phospho-NF-κB, NF-κB, phospho-eIF-2α, eIF-2α and PKR in HNECs treated with 0.1–10 μg/ml curcumin. The corresponding expression levels are shown as error bar graphs. Data are means ± SEM. *p<0.05 versus only RSV.

It is thought that the replication of RSV is closely associated not only with activation of NF-κB but also with phosphorylation of eIF-2α and expression of protein kinase R (PKR) in infected cells [8]. To investigate the detailed mechanisms of the prevention by curcumin of RSV replication, we performed Western blotting for phospho-NF-κB and phospho-eIF-2α as well as expression of PKR in HNECs infected with RSV with and without curcumin. The phosphorylation of eIF-2α after infection with RSV was enhanced by 10 µg/ml curcumin, but not by lower concentrations, whereas the phosphorylation of NF-κB after infection with RSV was decreased from 5 µg/ml curcumin (Figure 6). Expression of PKR was also increased by 5 µg/ml curcumin (Figure 6).

### Salbrinal affects replication of RSV, expression of tight junction proteins and production of proinflammatory cytokines in HNECs infected with RSV

**Figure 7:**
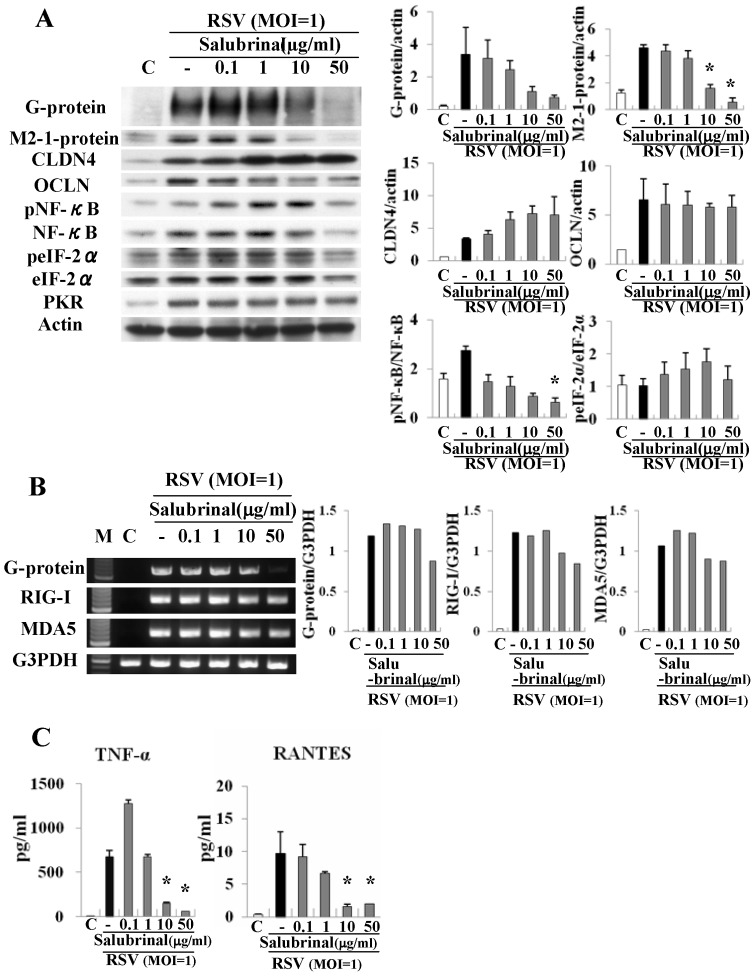
Western blotting (A) for G and M2-1 proteins, claudin-4, occludin, phospho-NF-κB, NF-κB, phospho-eIF-2α, eIF-2α and PKR, RT-PCR (B) for G protein, RIG-I and MDA5 and ELISA (C) for TNFα and RANTES in HNECs pretreated with 0.1–50 μg/ml salubrinal before infection with RSV at an MOI of 1. The corresponding expression levels are shown as error bar graphs. Data are means ± SEM. *p<0.05 versus only RSV. CLDN: claudin, OCLN: occludin.

To investigate whether the eIF-2α dephosphorylation inhibitor salubirinal affected the replication of RSV, expression of tight junction proteins, and production of the epithelial cell responses, HNECs were pretreated with 0.1–50 µg/ml salubrinal 30 min before infection with RSV at an MOI of 1 for 24 h. In Western blotting, expression of G and M2-1 proteins after infection with RSV was inhibited from 10 µg/ml salubrinal, and upregulation of phospho-NF-κB and phospho-eIF-2α after infection with RSV was decreased at 50 µg/ml salubrinal (Figure 7A). The upregulation of claudin-4, occludin and PKR after infection with RSV was not affected by any dose of salubrinal (Figure 7A). In RT-PCR, the production of mRNAs of G protein after infection with RSV was inhibited by 50 µg/ml salubrinal and expression of mRNAs of RIG-I and MDA5 after infection with RSV was inhibited from 10 µg/ml salubrinal (Figure 7B). By ELISA, upregulation of TNFα and RANTES production after infection with RSV was significantly inhibited from 10 µg/ml salubrinal (Figure 7C).

### MG132 affects replication of RSV, expression of tight junction proteins and production of proinflammatory cytokines in HNECs infected with RSV

**Figure 8:**
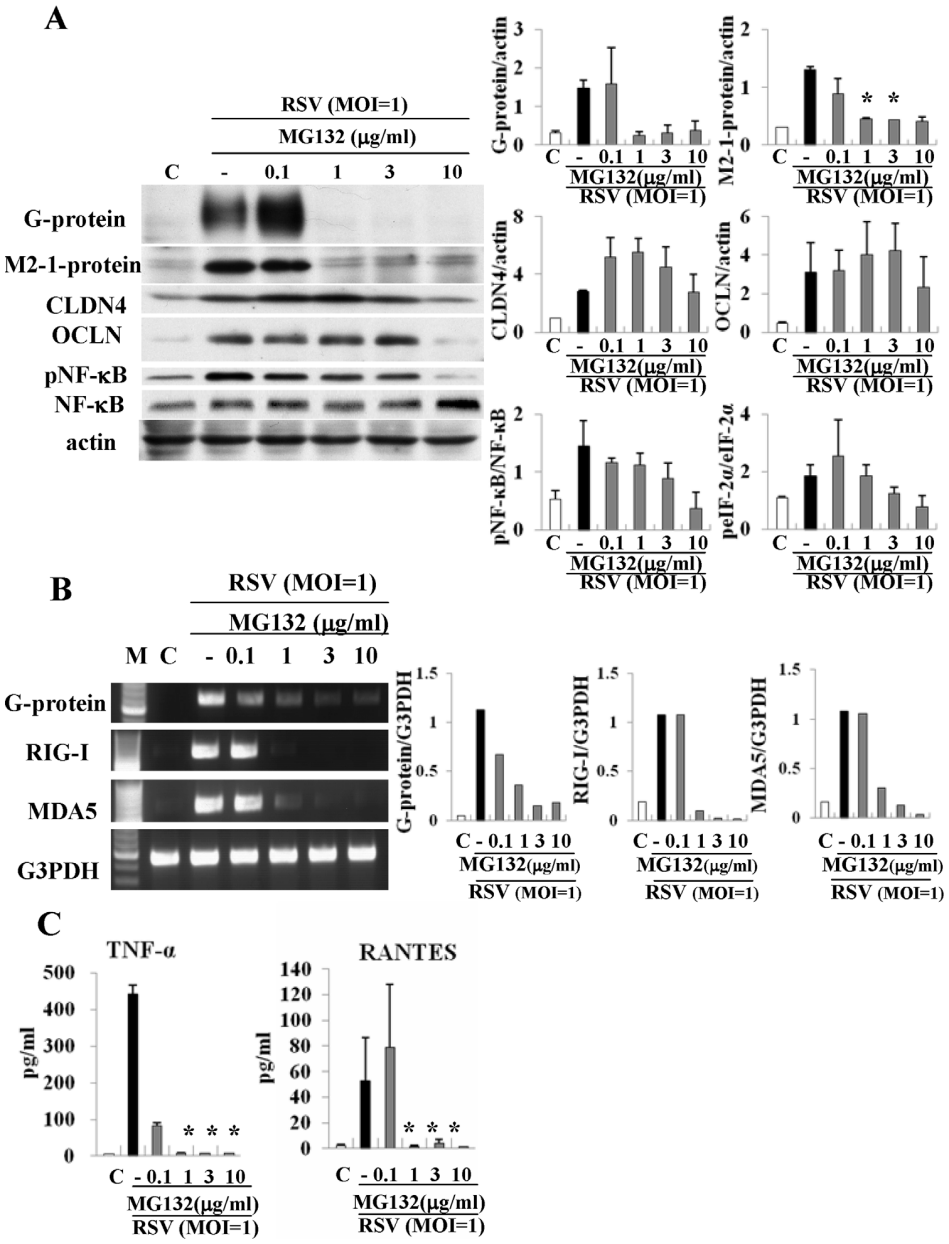
Western blotting (A) for G and M2-1 proteins, claudin-4, occludin, phospho-NF-κB and NF-κB, RT-PCR (B) for G protein, RIG-I and MDA5 and ELISA (C) for TNFα and RANTES in HNECs pretreated with 0.1–10 μg/ml MG132 before infection with RSV at an MOI of 1. The corresponding expression levels are shown as error bar graphs. Data are means ± SEM. *p<0.05 versus only RSV. CLDN: claudin, OCLN: occludin.

It is known that curcumin in part prevents activation of NF-κB through inhibition of proteasomes [35]. To investigate whether proteasome inhibitor MG132, which inhibits degradation of IκBα, affected replication of RSV, expression of claudin-4 and occludin, and production of proinflammatory cytokines, HNECs were pretreated with 0.1–10 µg/ml MG132 30 min before infection with RSV at an MOI of 1 for 24 h. In Western blotting, expression of G and M2-1 proteins after infection with RSV was inhibited from 1 µg/ml MG132, and upregulation of phospho-NF-κB, claudin-4 and occludin after infection with RSV was increased at 10 µg/ml MG132 (Figure 8A). In RT-PCR, upregulation of mRNAs of G protein after infection with RSV was inhibited from 0.1 µg/ml MG132 and upregulation of RIG-I and MDA5 was inhibited from 1 µg/ml MG132 (Figure 8B). In ELISA, upregulation of TNFα and RANTES production after infection with RSV was inhibited from 0.1 and 1 µg/ml MG132, respectively (Figure 8C).

### Inhibitors of COX1 and COX2 affect replication of RSV, expression of tight junction proteins and production of proinflammatory cytokines in HNECs infected with RSV

**Figure 9:**
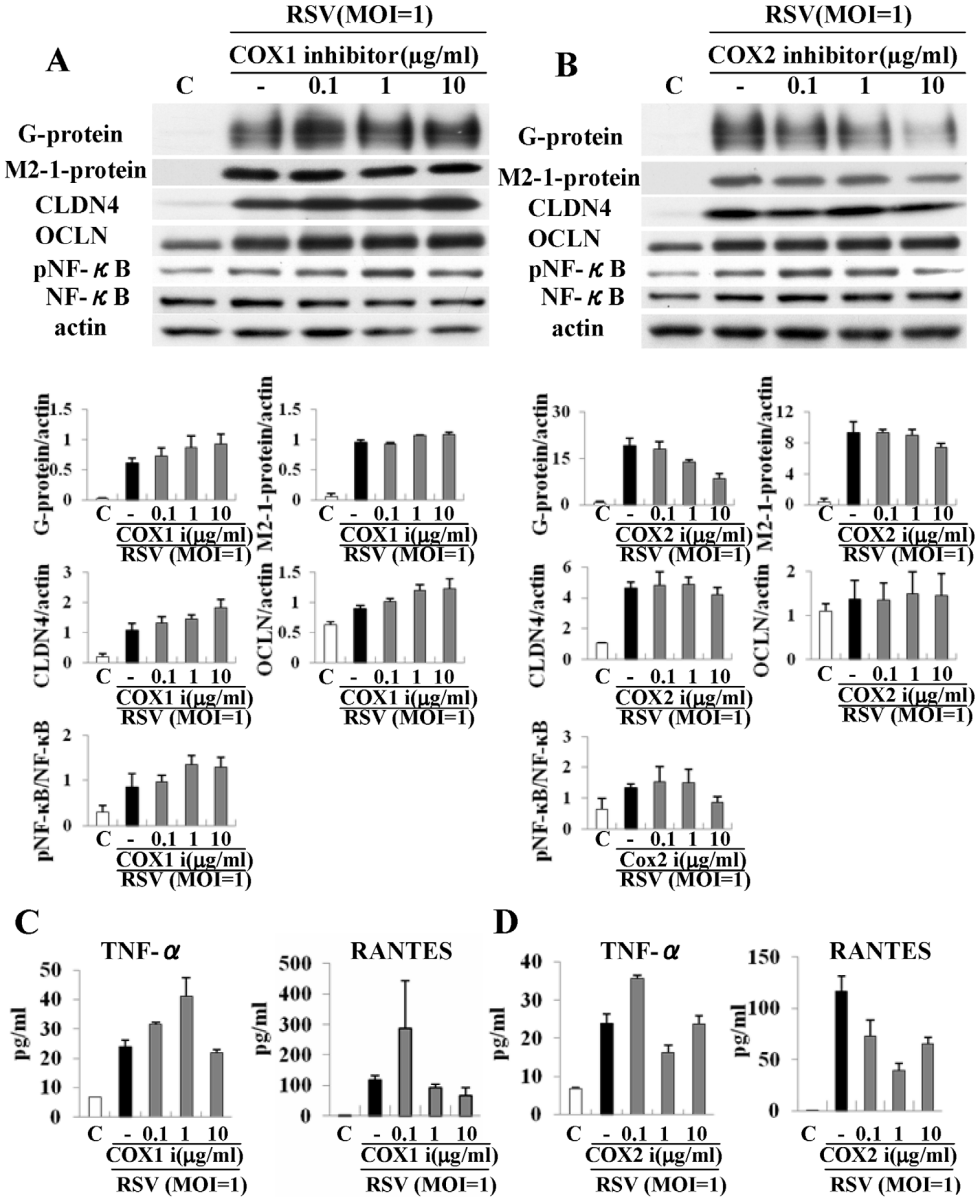
Western blotting (A, B) for G and M2-1 proteins, claudin-4, occludin, phospho-NF-κB and NF-κB, and ELISA (C, D) for TNFα and RANTES in HNECs pretreated with 0.1–10 μg/ml COX1 inhibitor or COX2 inhibitor before infection with RSV at an MOI of 1. The corresponding expression levels are shown as error bar graphs. Data are means ± SEM. *p<0.05 versus only RSV. CLDN: claudin, OCLN: occludin.

Curcumin has potential inhibitory effects against COX2 [26]. In the present study, GeneChip analysis showed that upregulation of COX-1 and COX-3 was induced by infection with RSV, and curcumin downregulated the expression of COX1, COX2 and COX3 after infection with RSV (Table 3). To investigate whether inhibitors of COX1 and COX2 could prevent RSV replication, up-regulation of claudin-4 and occludin, and expression of proinflammatory cytokines, HNECs were pretreated with 0.1–10 µg/ml both inhibitors 30 min before infection with RSV at an MOI of 1 for 24 h. In Western blotting, the expression of G and M2-1 protein and upregulation of phospho-NF-κB after infection with RSV were inhibited by 10 µg/ml of the COX2 inhibitor but not the COX1 inhibitor (Figure 9A, 9B). In ELISA, upregulation of RANTES production after infection with RSV was inhibited by 1 µg/ml COX1 and COX2 inhibitors and upregulation of TNFα production after infection with RSV was inhibited by the 1 µg/ml COX2 inhibitor (Figure 9C, 9D). The inhibitors of COX1 and COX2 did not affect upregulation of claudin-4 and occludin after infection with RSV in Western blotting (Figure 9A, 9B).

## Discussion

In the present study, we demonstrated that curcumin prevented the replication and budding of RSV, release of TNFα and RANTES and expression of RIG-I and MDA5 via its multiple functions in HNECs.

In our previous study, the NF-κB inhibitor IMD0354 inhibited replication and budding of RSV and release of proinflammatory cytokines in HNECs infected with RSV [11]. In the present study, when other NF-κB inhibitors, curcumin and PDTC, were used to treat HNECs infected with RSV, curcumin, but not PDTC, suppressed expression of G and M2-1 proteins and phosphorylation of NF-κB without cytotoxity. However, in the present study, curcumin did not affect replication of RSV in human lung adenocarcinoma cell line A549 infected with RSV. These results suggested that curcumin was more effective to prevent replication of RSV in HNECs than in lung epithelial cells.

The phosphorylation of eIF-2α is upregulated by curcumin in A549 cells [25]. RSV induces and activates PKR, a cellular kinase relevant to limiting viral replication, which regulates the activation of the translation initiation factor eIF-2α [8]–[10]. A selective inhibitor of eIF-2α dephosphorylation, salubrinal, has antiviral effects against Epstein-Barr virus (EBV) replication [36], [37]. In the present study, curcumin increased the phosphorylation of eIF-2α and expression of PKR in HNECs after RSV infection. Furthermore, in HNECs after RSV infection, salubrinal also prevented the replication of RSV and release of TNFα and RANTES with inhibition of phosphorylation of NF-κB and eIF-2a. These findings indicated that, in HNECs, curcumin might prevent replication of RSV and release of TNFα and RANTES via not only phosphorylation of NF-κB but also PKR/phosphorylation of eIF-2α, like salubrinal.

It is known that curcumin is a potent inhibitor of proteasomes [28]. The proteasome inhibitor MG132, which blocks activation of NF-κB by preventing proteasome-mediated degradation of IκB, inhibits replication of RSV in Vero cells [38]. In the present study, in HNECs infected with RSV, MG132 inhibited the replication of RSV, release of TNFα and RANTES and expression of RIG-I and MDA5. These results indicated that curcumin might prevent the replication of RSV, release of TNFα and RANTES and expression of RIG-I and MDA5 via a proteasome inhibitor like MG132.

RSV infection induces expression of COX2 but not COX1 [39]. In addition, COX2 is a potential therapeutic target in RSV-induced diseases in the human lung [40], [41]. In the present study, by GeneChip analysis of HNECs infected with RSV, upregulation of COX1 and COX3 was observed compared to the control, and downregulation of COX1, COX2 and COX3 was induced by treatment with curcumin. When HNECs infected with RSV were treated with an inhibitor of COX1 or COX2, COX2 inhibitor prevented the replication of RSV, phosphorylation of NF-κB and release of TNFα and RANTES. These findings suggested that curcumin might be a potent inhibitor of COX2 in HNECs and that the effects of the COX2 inhibitor played a crucial role to prevent the epithelial inflammatory responses to RSV infection.

Curcumin prevents disruption of tight junctions and the barrier induced by IL-1β or H_2_O_2_ in human intestinal epithelial cells [31], [42]. In the present study, expression of claudin-4 and occludin and the barrier function in HNECs were increased by treatment with curcumin (Fig. 2, data not shown). Furthermore, as demonstrated by Gene chip analysis, in RSV-infected HNECs without curcumin, upregulation of claudin-1, −3, −4, −9, and −12, occludin, cingulin, and MAGI-1 was observed compared to the control, whereas in RSV-infected HNECs with 5 µg/ml curcumin, upregulation of claudin-1, −4, and −12, occludin and cingulin was observed compared to RSV-infected HNECs without curcumin. The barrier function of HNECs was increased after infection with RSV and The barrier function of RSV infected HNECs was more enhanced by treatment from 5 µg/ml curcumin compared to RSV infected HNECs without curcumin. In Western blotting and RT-PCR, expression of claudin-4 and occludin in RSV infected HNECs was increased by treatment until 5 µg/ml curcumin and decreased by treatment with 10 µg/ml curcumin. The detailed mechanisms of the effects of curcumin for tight junction molecules in RSV-infected HNECs remain unclear.

**Figure 10:**
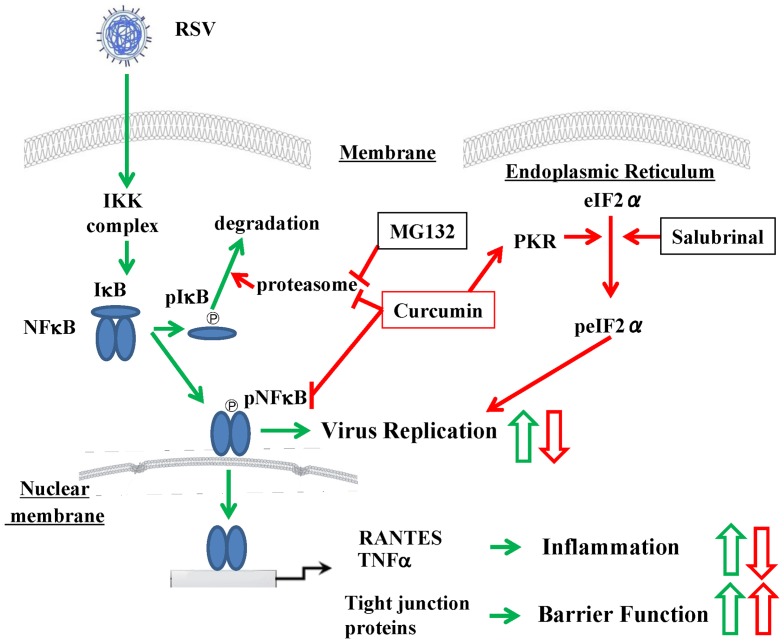
Overview of signal transduction events in RSV infected HNECs with and without curcumin, salubrinal and MG132. Closed green arrows: effects of RSV-infection, Open green arrows: upregulation, closed red arrows: effects of inhibitors, open red arrows: downregulation.

In conclusion, the cytotoxicity of curcumin was not observed at high doses in normal HNECs. Curcumin completely prevented the replication and budding of RSV and the epithelial responses in HNECs and strongly increased the epithelial barrier of HNECs via its pharmacokinetic effects (Figure 10). Inhibition of RSV in upper airway HNECs by curcumin may be effective for the prevention of severe lower respiratory tract disease in infants and young children.
